# Integration of transcript expression, copy number and LOH analysis of infiltrating ductal carcinoma of the breast

**DOI:** 10.1186/1471-2407-10-460

**Published:** 2010-08-27

**Authors:** Lesleyann Hawthorn, Jesse Luce, Leighton Stein, Jenniffer Rothschild

**Affiliations:** 1 Medical College of Georgia Cancer Center, 1120 15th St, Augusta, GA. 30912, USA; 2 Roswell Park Cancer Institute, 666 Elm St, Buffalo, NY 14263, USA

## Abstract

**Background:**

A major challenge in the interpretation of genomic profiling data generated from breast cancer samples is the identification of driver genes as distinct from bystander genes which do not impact tumorigenesis. One way to assess the relative importance of alterations in the transcriptome profile is to combine parallel analyses that assess changes in the copy number alterations (CNAs). This integrated analysis permits the identification of genes with altered expression that map within specific chromosomal regions which demonstrate copy number alterations, providing a mechanistic approach to identify the 'driver genes'.

**Methods:**

We have performed whole genome analysis of CNAs using the Affymetrix 250K Mapping array on 22 infiltrating ductal carcinoma samples (IDCs). Analysis of transcript expression alterations was performed using the Affymetrix U133 Plus2.0 array on 16 IDC samples. Fourteen IDC samples were analyzed using both platforms and the data integrated. We also incorporated data from loss of heterozygosity (LOH) analysis to identify genes showing altered expression in LOH regions.

**Results:**

Common chromosome gains and amplifications were identified at 1q21.3, 6p21.3, 7p11.2-p12.1, 8q21.11 and 8q24.3. A novel amplicon was identified at 5p15.33. Frequent losses were found at 1p36.22, 8q23.3, 11p13, 11q23, and 22q13. Over 130 genes were identified with concurrent increases or decreases in expression that mapped to these regions of copy number alterations. LOH analysis revealed three tumors with whole chromosome or p arm allelic loss of chromosome 17. Genes were identified that mapped to copy neutral LOH regions. LOH with accompanying copy loss was detected on Xp24 and Xp25 and genes mapping to these regions with decreased expression were identified. Gene expression data highlighted the PPARα/RXRα Activation Pathway as down-regulated in the tumor samples.

**Conclusion:**

We have demonstrated the utility of the application of integrated analysis using high resolution CGH and whole genome transcript analysis for detecting driver genes in IDC. The high resolution platform allowed a refined demarcation of CNAs and gene expression profiling provided a mechanism to detect genes directly impacted by the CNA. This is the first report of LOH integrated with gene expression in IDC using a high resolution platform.

## Background

Breast cancer is the most frequently diagnosed malignancy among women. In 2008, an estimated 184,450 new cases of breast cancer occurred in the United States and during that same year, it is estimated that almost 41,000 women died of breast cancer [1]. The most common type of breast cancer is infiltrating ductal carcinoma (also called invasive ductal carcinoma) (IDC), which accounts for approximately 80 percent of all breast cancer cases. Overall, these numbers reflect a reduction in breast cancer-related mortality due to improved screening and therapeutic options [2]. However, these statistics do not completely depict the innovation in the treatment perspectives that have occurred in the past decade. Particularly, the genomic era has been characterized by an exponential increase in the number of putative therapeutic targets by defining subtypes based on molecular profiles [3].

High-throughput molecular profiling resources permit an almost complete inventory of transcript expression or DNA copy number alterations in cancer specimens. However a major challenge in the biological interpretation of these vast data sets remains. The role of chromosomal copy number alterations (CNAs) in the neoplastic process is well documented. Genome-wide comparative genomic hybridization (CGH) has been used to profile IDCs in a large number of studies [4]. These studies have suggested recurrent gains at 1q31-q32, 8p12, 8q12 and 8q24, 11q13, 17q12, 17q23-q24, and 20q13, recurrent losses are observed at 1p, 6q, 8p, 11q23-qter, 13q, 16q, 17p and 22q [5].

With the emergence of array-based CGH (aCGH) technologies it is now possible to resolve regions of genomic CN gain and deletion at ultra high resolution. In addition to improved resolution, we are also able to incorporate statistical methods to identify novel regions of loss or gain that correlate to known CN gains or deletions. We have used Affymetrix 250K Mapping arrays to profile the genome of 22 infiltrating ductal breast tumors at a 5.8 Kb resolution. One major advantage of our approach is that the SNP arrays can also identify loss of heterozygosity events that result from all genetic events that give rise to LOH, even in the absence of a CNA. LOH is expected to expose recessive mutations in critical genes in the genomic regions defined by the LOH.

Gene expression profiling using microarray analysis has shown to be a powerful tool to predict tumor behavior. It has been shown that using the gene expression profile of the tumor, prognosis can be more accurately predicted than by clinical variables alone. One way to assess the relative importance of gene expression changes is to combine complementary analyses from the same biological samples that assess changes in the physical genomic profile. This type of integrated analysis can potentially identify genes within specific chromosomal regions that demonstrate CNA with corresponding increases or decreases in gene expression, thereby providing a filter to determine the 'drivers' of the CNA. Recently several reports have described this integrated approach to the analysis of breast cancer [6-14]

Here we report the analysis of CNA in a series of frozen, micro-dissected IDC specimens. We have used 14 of the same samples to perform transcript expression analysis. We have then integrated these two high resolution data sets to identify regions of consistent copy number alterations and the genes that map to these regions that simultaneously display transcript expression alterations. Using assimilation of these two whole genome analyses approaches, we have identified genes showing up or down regulation which map specifically to regions of copy number gains or losses. We also incorporated data from LOH analysis to identify genes showing altered expression in LOH regions.

## Methods

Twenty three frozen breast samples were chosen for analysis. All tumors were obtained under Institutional Review Board approved protocols. Twenty one of these were IDCs and 2 were metastatic lymph node biopsies. All tumors were fresh frozen, micro-dissected and assessed by a pathologist to assure that over 80% of the cells present were neoplastic. Clinical features of the samples are presented in table 1. For gene expression analysis, 4 normal control samples were obtained from tissues adjacent to tumors of 2 patients used in the study and 2 from adjacent tumors obtained from patients not included in the study (see patient numbers Table 1)

**Table 1:** Clinical information and array platform used for each sample.

Patient Number	Tissue	Diagnosis	Experiments/Arrays
1	breast tumor	poorly differentiated infiltrating ductal carcinoma	250K, 133plus2.0
2	breast tumor	infiltrating ductal carcinoma	250K, 133plus2.0
3	breast tumor	infiltrating carcinoma	250K
4	breast tumor	infiltrating ductal carcinoma	250K, 133plus2.0
4	non-tumor breast	adequate control tissue, T-0400, NOS	133Plus2.0
5	breast tumor	infiltrating ductal carcinoma	250K, 133plus2.0
6	breast tumor	poorly differentiated infiltrating ductal carcinoma	250K, 133plus2.0
7	breast tumor	infiltrating ductal carcinoma	250K, 133plus2.0
8	breast tumor	infiltrating ductal carcinoma	250K, 133plus2.0
9	Lymph node	metastatic ductal carcinoma to lymph node	250K
9	breast tumor	infiltrating ductal carcinoma	250K
11	breast tumor	infiltrating ductal carcinoma and high grade DCIS	250K
12	breast tumor	infiltrating ductal carcinoma	250K, 133plus2.0
13	breast tumor	infiltrating ductal carcinoma	250K, 133plus2.0
13	non-tumor breast	adequate control tissue, T-0400, NOS	133Plus2.0
14	breast tumor	infiltrating ductal carcinoma	250K
15	breast tumor	infiltrating ductal carcinoma	250K
16	Lymph node	metastatic ductal carcinoma to lymph node	250K, 133plus2.0
17	breast tumor	Invasive ductal carcinoma	250K
18	breast tumor	Invasive ductal carcinoma	250K, 133plus2.0
19	breast tumor	Invasive ductal carcinoma	250K
20	breast tumor	Invasive ductal carcinoma	250K, 133plus2.0
21	breast tumor	Invasive ductal carcinoma	250K
22	breast tumor	Invasive ductal carcinoma	250K, 133plus2.0
23	breast tumor	Invasive ductal carcinoma	133Plus2.0
24	breast tumor	Invasive ductal carcinoma	133Plus2.0
25	breast tumor	Invasive ductal carcinoma	133plus2.0
26	non-tumor breast	adequate control tissue, T-0400, NOS	133Plus2.0
27	non-tumor breast	adequate control tissue, T-0400, NOS	133Plus2.0

### Copy Number Analysis using SNPArray CGH

The Affymetrix GeneChip 250K Mapping Assay is designed to detect > 250,000 Single Nucleotide Polymorphisms (SNPs) in samples of genomic DNA. Array experiments were performed previously described [15]. Briefly, 250 ng of genomic DNA was digested with the restriction enzyme STY. The assay utilizes a strategy which reduces the complexity of human genomic DNA up to 10 fold by first digesting the genomic DNA and then ligating STY adaptor sequences onto the DNA fragments. The complexity is further reduced by a PCR procedure optimized for fragments of a specified size range (200-1100 bp). Following these steps the PCR products (amplicons) are fragmented, end-labeled, and hybridized to the array.

### Copy Number Data Analysis

**Figure 1:**
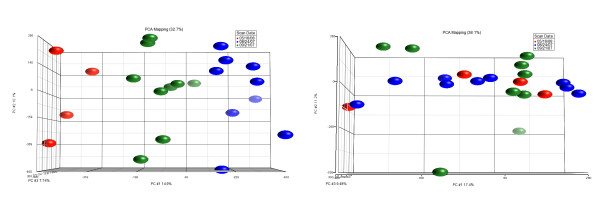
**Principle Components Analysis of Copy Number Data**. Principle Components Analysis PCA is a method of dimensionality reduction to look for overall trends in the data. In figure 1a it can be seen that "scan date" has had a significant impact on the data implying that processing of the samples at different times has contributed significantly to the variance in the samples meaning that this significant contribution may obscure the main effects. Figure 2b shows the PCA plot following batch removal of the effect of Scan Date. A mixed model ANOVA was used to estimate the effect of scan date and removed from the data.

Following the washing staining and scanning the .CEL files generated from Affymetrix Command Console were transferred to PARTEK Genomics Suite 6.5. We first adjusted the raw probe intensities based on the GC content of the sequence. This correction has been shown to improve the accuracy of CNA calls [16]. This adjustment was followed by probe-level normalization of signal intensity while simultaneously adjusting for fragment length and probe sequences across all samples. The data were then background corrected using RMA and quantilie normalized. The baseline was generated from the 250K Mapping 270HapMap set obtained from CEPH (Centre Etude du Polmorphisme Humain) individuals. Overall quality assessment was performed using Principle Components Analysis (PCA) (Figure 1). A 2- way analysis of variance (ANOVA) was then performed on the data using tissue type and scan date as variables. Scan date showed significant contribution to the PCA (see figure 1) and therefore was removed as a batch effect (figure 1b). The resultant data was then used to generate CNA for each sample. Detection of CN gains and deletions was performed using the Genomic Segmentation algorithm available in PARTEK Genomics Suite to obtain the different CN state partitions. This algorithm is similar to HMM segmentation but instead of searching for regions from a specified list of states, the Genomic Segmentation Algorithm finds breakpoints in the data. This algorithm has several advantages over HMM. Cancer derived samples are likely to contain different populations of cells, which may not display the same copy number variations. For this reason, copy number often will not fall into biologically predicted bins and occasionally become continuous variables. Segmentation looks for changes in genomic abundance, not regions of a specific copy number state, enabling segmentation to be highly effective in cases where tissue heterogeneity can lead to non-integer copy number intensities. The Genomic Segmentation Algorithm does not bin the regions into predefined states; instead regions will be called with a mean at any copy number state with no redefined normal bin filtered out. The different segments are then defined as regions of locally stable copy number states and each region is compared to the expected normal value and assigned a likelihood of being a CNA using two one-sided t-tests. The resultant p-values are used to filter out regions of change that are rare or due to noise. Noise is significant in copy number data so that the algorithm does not consider normal at a diploid number of 2 but instead is considered a range of+/-2.3. Therefore, cutoff values of 2.3 for gains and 1.7 for losses were used and amplifications were defined as states exceeding 4.5 copies

We specified that each segment must contain a minimum of 10 consecutive filtered probesets. The 250K Mapping array has SNP probes placed average distance of 10 KB between probes therefore the copy number segments we are identifying are a minimal size of 100 KB, Although, in practice, this will not be the case on every chromosomal region because the marker density varies significantly across the genome. A threshold p-value of p = 0.001 for two adjacent regions having significantly different means using a two sided t-test was implemented. The signal to noise ratio was set at 0.3 and is estimated by the calculation of local estimates of standard deviation to determine if probes differ from neighboring probes across all samples. This estimate determines how robust the algorithm will be when applied to samples with highly variant genomes. The signal to noise setting is the minimum difference between two potential consecutive settings divided by the chromosomal variant estimate. We specified that the CNA had to occur in at least 5/24 samples.

### LOH analysis

For analysis of LOH events the raw image data from the 24 samples was incorporated into Genotyping Console (Affymetrix lnc., CA, USA) which automatically generates genotype calls using the Bayesian Robust Linear Model with Mahalanobis (BRLMM) distance classifier algorithm http://media.affymetrix.com/support/technical/whitepapers/brlmm_whitepaper.pdf. A genotype and confidence score is assigned for each observation. The resultant .CHP file contains calls (AA, BB or AB) for each SNP probe set. The .CHP file was then imported into PARTEK Genomics Suite ver 6.4. PARTEK analysis of LOH uses a Hidden Markov Model (HMM) to find regions that are most likely to be loss events based on the genotype error and the expected heterozygous frequency at each SNP. We used an unpaired analysis where the probability of observing a heterozygous SNP in a region of LOH is the genotype error rate. In a region without LOH, the probability of observing a heterozygous SNP is estimated using the observed frequency from the baseline samples. The heterozygosity rate (HET rate) is calculated as the number of AB calls/total number of calls, therefore low het rates imply LOH. By default the frequency of heterozygous calls in a normal region is .3. We used a het rate of <0.07 for detecting LOH events. The allelic ratios for the SNPs at each reported event were graphed and visually examined and any reported regions that were found in areas of poor probe density or close to centromeres were identified. We report only those LOH events which occurred in 3/24 samples. The analysis limits the number of markers on the LOH fragment to a minimum of 10. The LOH data was then assimilated and compared with copy number and with gene expression data.

We also analyzed the data using PennCNV [17] for comparison. This algorithm generates data listing 6 states of copy number events. A log R ratio (LRR) which is a measure of normalized and total signal intensity is calculated along with the B-allele frequency which is a measure of normalized allele intensity.

### Gene Expression Analysis

**Figure 2:**
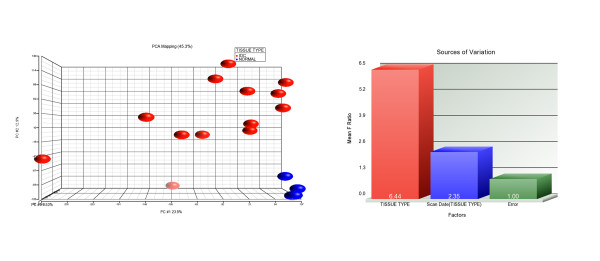
**PCA and ANOVA of Transcript Expression Data**. Analysis of Variance (ANOVA) was performed on the entire data set and a gene list was generated Figure 2a shows the histogram of variance indicating that the major variance was due to the differences between tumor and normal samples and scan date variance is almost the same as that attributed to type 1 error. PCA was used reduce dimensionality and to examine whether clusters of tumors separated from normal samples or if other variables, such as scan date contributed to the variance. It can be seen that the tumors are spread throughout the three dimensional space of the plot while the normal samples form a tight cluster at the base of the the plot on the right. Concordance of the clustering structure was observed between the two dimensionality-reduction procedures (data not shown). Analysis of Variance (ANOVA) was then performed on the entire data set and a gene list was generated using an FDR of 0.05, 2-fold expression changes and a p-value for fold change of 0.05. Figure 2 shows the histogram of variance and the PCA plot of the effect of the scan date on the data.

RNA obtained from the tumors and accompanying normal tissues was used to prepare cRNA for hybridization to the Affymetrix U133Plus 2.0 oligonucleotide arrays as described previously [15]. This analysis included 16 IDC samples and 4 control breast tissues. All procedures were carried out as specified by the manufacturer. Following hybridization to the U133Plus 2.0 arrays, the resultant raw CEL files were transferred to PARTEK Genomics Suite version 6.5 and normalized using GCRMA with quantile normalization to correct for variances in distribution patterns and GC nucleotide content. After performing the normalization, a PCA was used reduce dimensionality and to examine whether clusters could be explained by the first few principle components, which are ordered by the Eigen values of the covariance matrix. Analysis of Variance (ANOVA) was then performed on the entire data set. Figure 2 shows the histogram of variance and the PCA plot of the effect of the scan date on the data. A gene list was then generated using an FDR (Benjamini Hochberg) [18] of 0.05 and a 2 fold cut off for fold change. Figure 2 shows the histogram of variance and the PCA plot of the effect of the scan date on the data.

To assess the possible functional connections between the differentially expressed genes (DEGs), a pathways analysis, which assesses statistically overrepresented functional terms within a list, was conducted using Ingenuity Pathways Analysis (Ingenuity Systems^®^) (IPA). The probability that a specific set of genes has a significant number of members in a canonical pathway is assigned a p-value which is calculated by Fisher's Exact Test (right tailed). The p-value indicates the probability of observing the fraction of the focus genes in the canonical pathway compared to the fraction expected by chance in the reference set, with the assumption that each gene is equally likely to be picked by chance.

### Integration Analysis

Using PARTEK Genomics Suite, an analysis was performed on 14 samples with complementary datasets from both SNP-CGH and expression profiling. Each sample is analyzed for overlapping events in copy number and gene expression analysis. For the Affymetrix GeneChip U133A platform, an FDR of 0.05 and 2-fold expression changes and was used evaluate either up- or down- regulation of gene expression. Copy number alterations were defined as <1.3 (loss) or >2.3 (gains). Concordant changes in SNP-CGH and gene expression as defined above (i.e. chromosomal gain with up-regulation of gene transcript and vice versa) were calculated for each tumor. For an overlay event we specified the loss/under-expression (or gain/over-expression) had to occur in at least 3 of the 14 overlapping data sets.

## Results

### Copy Number Analysis

**Figure 3:**
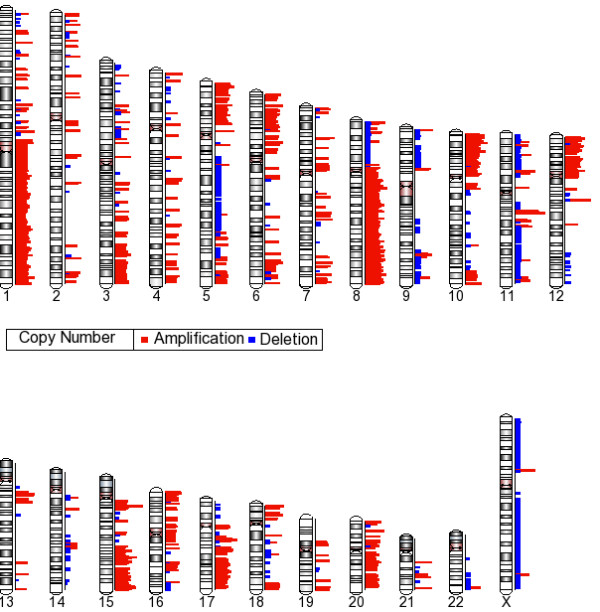
**Copy Number Alterations in Infiltrating Ductal Carcinoma**. Copy number data was generated using Genomic segmentation to obtain the different CN state partitions. The baseline was the HapMap 270 set of samples. The gains/losses are shown in red and blue respectively for each chromosome. Each copy number event was required occur in 5/22 samples. The cutoff values for copy numbers of 1.7 or less for deletions and 2.3 or greater for CN gains. The length of each bar represents the average copy number across all samples. The range is from -2 to 22.

Following Genomic segmentation to obtain the different CN state partitions, the gains/losses and CN amplifications were identified. The CNA was required occur in at least 5/24 samples. Our final data summarization used cutoff values for copy numbers of 1.7 or less for losses and 2.3 or greater for CN gains. Figure 3 shows the ideogram of the copy number gains and losses. The length of each bar represents average copy number. Using these criteria, we found 6011 discreet regions of CN gains (3903) and losses (2018). Virtually every chromosome has regions of CNA.

Amplifications were defined as the more than 4.5 copies. A 5 MB amplification at 13q34 (13:108610073-11406382) had 9-14 copies in 5-6 samples. Another 7.3 MB amplification was detected at 11q13 (11:68276247-70252778) in 5-10 samples with copy numbers ranging from 5-7. Other amplified regions included 1p12, 1p13, 1q44, 4q22.3, 5p12, 5p15.33, 6p21.1, 6p24.3, 7p15.2, 8p23.3, 8q12.1, 8q21.3 8q23.3, 8q24.11-q24.13, 9p24, 10p13, 10p14, 10p15.1-p15.3, 11p13, 12p13.31, 12q14.1, 15q12, 15q14.1, 15q24.1, 15q26.3, 17q12, 18p11.32, 20p12.3 and Xp11.3.

**Figure 4:**
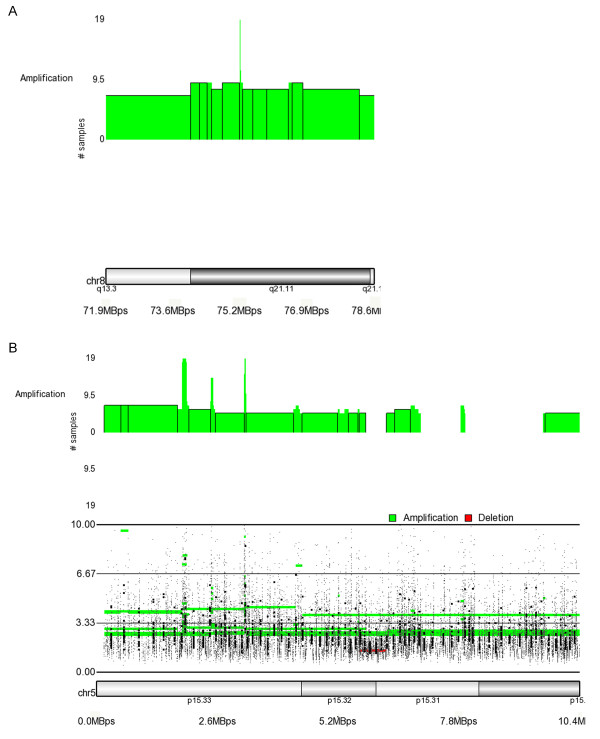
**Minimal Regions of Overlap in Copy Number**. A amplified region on chromosome 8q21.11 is shown in 4a. The minimal region of overlap has an average of 4.9 copies in 19 samples. This small region is 13 KB (8: 75237536-75250960) and is surrounded by an amplification or gain in 7-18 samples. This extended region is 90 KB and begins at 8p11 and extends to the telomere (8:41489634- 133808163). 4b shows an novel copy number region of gain at 5p15.33 where two minimal regions of overlap were detected. The first extends 101 KB (5:1836735-1938410) and the second is 19 KB (5:3180419-3199829) while the entire amplified region extends 5.6 MB from 5p15.33-p33.2 (5:165712-5779631). The bottom trace shows the SNP intensity plot for the region. The larger dots are smoothed data and the fine dots are unsmoothed. The first region has a mean of 3.5 copies and the second has 4.7.

High frequency gains were detected at 5p15.33, 8q21.11, 6p21.3, 7p11.2-7p12.1, 8q24.3 and 1q21.3. The regions with gains or amplifications in the largest number of tumors, defined as the minimal region of overlap, are often small and flanked by adjacent regions where the CN gain region is extended in size in a fewer number of samples. This is shown in figure 4a where a small amplification of 4.9 copies on chromosome 8q21.11 was detected in 19 samples and this region is surrounded by an amplification or gain in a fewer number of tumors. Another region of amplification on chromosome 8q24.3 (8:142253102-142791192), was .53 MB in size with an average copy number of 5 in 17/24 tumors. This represents the most frequently amplified region in a much larger region of CN gain extending from 8q24.22-q24.3 (8:137928635-146264219) an 8.3 Mb region in 5-17/24 samples. A similar situation exists for a copy number gain on chromosome 1q32 in18/24 tumors. The .2 Mb gain (1:199059280-200016637) is extended to a 15 Mb (1:198105319-213121513) contiguous gain in 5-18 tumors. A frequent gain was detected at 17q22-q23.2 (17:53263663-57020608) but the smallest region of overlap was defined at 17q23.3 (56639372-56770843) in 14 tumors. This analysis demonstrates the utility of the SNP array in pinpointing small regions of CN gain at a high resolution.

A region not commonly associated with breast cancer was identified showing amplification or gain in the largest number of tumors. There were two minimal regions of overlap in 19 tumors at 5p15.33 while the entire gain extends to the 5p15.32 as shown in figure 4b.

In general, fewer tumors carried common copy number losses. We set the homozygous deletion rate at <.5 average copy across samples. Using this approach we did not detect any homozygous deletions. Using the PennCNV algorithm, several were detected, however, they were detected in fewer than 2 samples and in most cases less than 10 SNPs were mapped to the fragments. Chromosome 11q22.3 showed a homozygous deletion in tumors 21 and 24 but no genes map to the region. Another deletion was detected at 11q23.1 was detected in tumors 1 and 3, however this fragment was only 15 KB and again, no genes were mapped to the region. Other homozygous deletions were detected at 11q14.2, 12q21.31 and 3p24.1. The small size and few SNPs on the fragments indicates that these may be artifacts of processing.

The highest frequency loss in 14/24 tumors at 11q23 (11:116268571-1116440064) is the minimal region of overlap and is extended to a larger region by the loss in a fewer number of tumors. Other regions of frequent regions of loss included; 1p36.22, 9q21.32, 11p13, 15q14, and 22q13.2 in 13-14/24 tumors. Loss at 8p is commonly identified and our analysis defined 3 large regions of loss including 8p23.3-p23.2 (8:716948-4795012), 8p23.3-p23.1 (8:4845891-9209368) and 8p23.1-p21.1 (8:9277842-30642261). These large contiguous regions also contained smaller minimal regions of overlap. The copy number data is available in Additional file 1, Table S1.

The CNAs that were detected were compared to copy number variant regions (CNVR) reported in The Database of Genomic Variants. http://projects.tcag.ca/variation/ng42m_cnv.php. In many cases CNVRs map into our larger regions of copy number alterations but do not constitute the entire CNA. In one instance, however, a copy number gain detected at chromosome 4p16.1, (14:9690464-9733791) overlapped significantly with a reported CNVR 1819.4 (14:9708917-9843664). No further information on this CNVR is available as it was not further investigated using the HapMap 470 samples therefore there are no estimates of copy number available at this loci.

### Loss of Heterozygosity LOH

**Figure 5:**
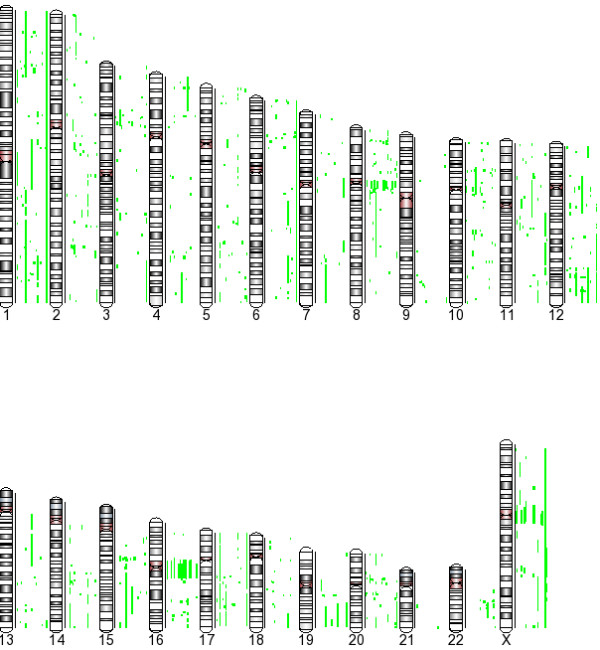
**LOH regions**. The plot shows LOH detected across the genome in 22 IDC samples. The genotype calls were generated using the BBRLMM algorithm. HMM was used to isolate those regions with a high probability to be loss events based on the genotype error and the expected heterozygous frequency at each SNP. The tumor numbers have the largest numbers closest to the chromosome. It can be seen that tumor 1, the furthest from each chromosome shows many regions of LOH including whole chromosome allelic loss of 1,6 and 17.

**Figure 6:**
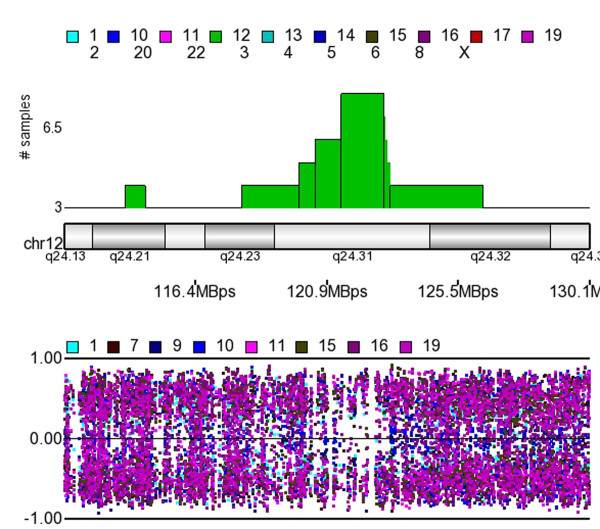
**Allelic Ratio Plot of 12q24.3**. The upper section of the figure plots the number of samples displaying LOH. The lower part of the diagram shows the allelic ratios plotted for each of the tumors defined by the colors shown above the plot. It is notable that the spots migrate towards 1 (AA calls) and -1 (BB calls) when the frequency of the LOH is increased, and as the frequency decreases, the more spots are plotted towards 0 (AB calls).

The allelic ratio plots for the SNPs at each reported event were visually examined and any that were found in regions of poor probe density or close to centromeres were excluded. For example, regions on 16p11.2 and 16q11.2 bordered the centromere and were categorized as amplified with LOH, however visual inspection suggested that these were SNP-poor regions and these were excluded from further analysis. A similar situation existed at 8q11.1 where there was a paucity of SNPs in the region making it difficult to validate the LOH event. The regions of LOH that met our criteria of arising in at least 3 samples, with fragments containing > 10 LOH markers and greater than 20 KB in length are shown in figure 5 where each horizontal bar represents a single tumor. Several tumors show whole chromosome LOH, for example tumor 1 has whole chromosome LOH on chromosomes 1, 6 and 17. Tumor 19 also shows LOH of all chromosome 17 while tumor15 shows loss of the q arm. A frequent LOH event on chromosome 12q24.31 (12:121425794-122926070) is shown in detail in figure 6. The allelic ratio plot shows the migration of the allelic ratio away from the central line 0 which represents AB calls towards the -1 and 1 grid lines which represent AA and BB calls. Additional file 2, Table S2 lists all the LOH events and details regarding the samples numbers and fragment length.

### Intregration of Copy Number with LOH

**Figure 7:**
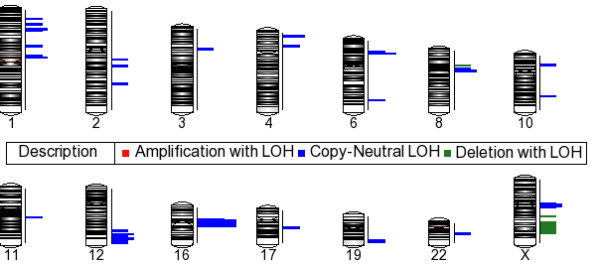
**Integration of Copy Number and LOH**. The integration was performed using the data generated using the criteria outlined for LOH and CNA described in the Materials and methods. The length of the bars indicates the number of samples. The LOH events close to the centromeres of chromosomes 8, 16 and X are the most frequent LOH events but not certifiable due to few probes in those regions. Copy number loss with LOH is detected at 8p12, Xp24 and Xp25. The other detected regions are copy neutral events.

The assimilation of copy number and LOH data determines if regions of LOH overlap with regions of deletion or amplification or are copy neutral events. The criteria defined above for selecting LOH and copy number regions were also used in this analysis. Our integration analysis determined that most LOH events were copy neutral. Deletions with LOH were detected only on several regions of the X chromosome and a region at chromosome 8p12. No regions of amplification accompanied by an LOH event were detected. The integration plot is shown in figure 7, with the length of the bars representing the number of tumors carrying the LOH event

We compared the data from PARTEK and PennCNV. Copy number and LOH analysis using PennCNV generally called fewer regions of CNA or LOH as previously reported [19]. The states defined by the PennCNV algorithm were generally similar to those defined by PARTEK so that (state 1) 0 copies, corresponded to homozygous deletions, (state 2) 1 copy, corresponded to LOH with deletion, (state 5) 3 copies single copy duplication defined in PARTEK by CN gain with LOH, and (state 6) 4 copies double copy duplication, also defined in PARTEK as CN gain with LOH. The PennCNV dataset is available in Additional file 3 Table S3. As a final output, it does not report copy neutral LOH which is important in studies of cancer. The algorithm has been designed to work primarily with Illumina Bead Studio data and is difficult to compare multiple tumors using Affymetrix data.

### Gene Expression

Following ANOVA, a gene expression difference (GED) list was generated using an FDR of 0.05 and expression alterations >2-fold increases or decreases. Figure 2b shows the PCA analysis of the IDC compared to the control samples. It can be seen that the controls from a very discreet cluster while the tumors are more spread across the different dimensions of the plot. Figure 2a shows a plot of the various sources of variance associated with ANOVA of the data and it is notable that the major source of variance is attributable to the differences between the two tissue types and the variance associated with the scan date is negligible and very close to the type 1 error. The analysis provides a list of 918 genes with altered expression between the tumor and control tissues. The majority of the alterations were 741 genes with decreased expression in the tumor tissues. The genes showing the highest levels of decreased expression were GPD1, KLB, SCL19A3, GLYAT, DGAT2, HSPB7, LVRN, CRYAB, S100B and MRAP. Of the 177 genes displaying increased expression those showing the highest expression included; ERBB3, KRT18, RAB25, PRSS8, SPINT2, BSPRY, CD24, SOX4, CLDN3 and EPB41L5. The entire DEG list is available as Additional file 4, Table S4. The raw data files for both SNP arrays and expression arrays are available in GEO accessions GSE22839 and GSE22840.

The data was then analyzed using IPA version 7.5. The canonical pathway that had the largest number of genes from our data contributing to it (18/158 members) (p = 1.6E-05) was the PPARα/RXRα Activation shown in Additional file 5, Figure S1. It can be seen that all of the members of this pathway that are target genes from our gene expression analysis show decreased expression in the tumor tissues (shown in green) indicating that the pathway is down-regulated. The second canonical pathway identified by our data was Prolactin Signaling followed by the PDGF Signaling Pathway.

### Copy Number and Gene Expression Integration Analysis

Using PARTEK Genomics Suite, an analysis was performed on 14 samples with complementary datasets from both SNP-CGH and expression profiling. Each sample is analyzed for overlapping events in copy number and gene expression analysis. For the Affymetrix GeneChip U133A platform, signal log ratios (SLR) of gene expression were generated for each tumor via comparison normal tissues as a group. Copy number alterations were defined as <1.3 (loss) or >2.3 (gains). Concordant changes in SNP-CGH and gene expression as defined above (i.e. chromosomal gain with up-regulation of gene transcript and vice versa) were calculated for each tumor. For an overlay event we specified the loss/under-expression (or gain/over-expression) had to occur in at least 3/14 overlapping data sets. One hundred and twenty nine genes showed altered expression levels and mapped to regions that showed corresponding chromosomal loss/gain. The results are shown in table 2 where copy number regions are shown with corresponding genes showing expression differences which have been detected in >5 tumors. The entire dataset is presented in Additional file 6, Table S5. For each tumor showing a CNA, individual expression values were examined and are presented in Additional file 6, Table S5 where the expression values are converted to standardized gene expression values for normal and tumor samples. These values were obtained by subtracting the probe set average across all tumors from each expression value. 'Negative numbers indicate that the gene expression value is lower than the average and positive ones indicate higher expression.

**Table 2:** Integration of copy number with gene expression data in > samples

Cyto-band	Gene Symbol	p-value	Fold-Change	length (bps)	Copy Number Location	Copy state	# Samples	Mean copy #
1q21.2	SV2A	0.000790217	2.35	1228718	chr1.147361719.148590436	gain	6	2.93
1q21.2	LOC730631	0.000454988	2.12	1228718	chr1.147361719.148590436	gain	6	2.93
1q21.3	S100A14	0.00103078	13.49	145569	chr1.151766410.151911978	gain	6	3.27
1q22	MUC1	3.86E-05	5.12	1636566	chr1.152807193.154443758	gain	6	2.79
1q23.2	IGSF9	1.70E-06	6.69	82022	chr1.158101327.158183348	gain	5	2.75
1q25.2	TDRD5	5.76E-08	2.49	76749	chr1.177855168.177931916	gain	6	3.12
1q32.1	PCTK3	0.000647034	2.12	57388	chr1.203711819.203769206	gain	7	2.12
1q41	CAPN8	5.28E-05	7.01	1214960	chr1.221390196.222605155	gain	6	2.76
1p34.1	ST3GAL3	0.00102362	-3.50	52158	chr1.44004795.44064981	loss	5	1.14
11p13	PRRG4	1.96E-07	4.61	129389	chr11.32799917.32929305	gain	7	3.42
11p11.2	PACSIN3	0.000775686	2.11	271627	chr11.47122445.47394071	gain	5	3.48
14q24.1	WDR22	5.46E-09	-2.06	2588491	chr14.68430400.69090879	loss	5	1.49
15q23	Hs.655686	5.25E-06	11.48	1692519	chr15.68908327.69703941	gain	5	2.58
15q23	Hs.655868	0.000235216	3.68	504419	chr15.69797809.70302227	gain	6	2.61
15q24.1	NEO1	0.00012075	2.11	843811	chr15.70912294.71756104	gain	7	3.37
15q26.1	ISG20	1.92E-06	3.16	249550	chr15.86957456.87207005	gain	6	2.84
15q26.1	FAM174B	0.000142779	4.64	127979	chr15.90953124.91081102	gain	5	3.50
15q26.1	FAM174B	4.31E-07	3.39	127979	chr15.90953124.91081102	gain	5	3.50
5p15.33	PLEKHG4B	0.000905578	2.33	352294	chr5.165712.518005	gain	5	3.26
6p24.3	TFAP2A	0.000412702	28.92	61721	chr6.10487807.10549527	gain	7	4.42
8q23.3	TRPS1	0.000200736	4.98	1434519	chr8.116140217.117574735	gain	5	3.65
8p22	DLC1	7.07E-05	-6.72	6333283	chr8.11614644.13945615	loss	6	1.49
8p21.3	GFRA2	1.72E-10	-2.18	1541653	chr8.20297943.21825688	loss	6	1.38
8p21.2	EBF2	5.96E-08	-4.59	61487	chr8.25917207.25978693	loss	5	1.46
8p21.2	AK057935	0.000340165	-8.34	798818	chr8.26553995.26777510	loss	6	1.47
8p12	Hs.654357	3.39E-06	-3.64	1469584	chr8.29124128.30492208	loss	5	1.48
8q12.1	SDR16C5	0.00075179	7.97	689931	chr8.56762001.57376988	gain	5	2.99
8p23.1	ANGPT2	2.01E-06	-6.11	168439	chr8.6342789.6444367	loss	5	1.45
8p23.1	DEFA1 /// DEFA3 /// LOC728358	6.87E-05	-3.05	1406779	chr8.6789275.6889920	loss	6	1.40
8q21.13	CHMP4C	0.000234239	16.46	5693518	chr8.81713285.87406802	gain	5	3.22
Xq22.3	Hs.715776	2.99E-07	-9.24	16623258	chrX.102883803.119358070	loss	5	1.52
Xq22.3	LOC100130886 /// TMEM164	0.000421953	-2.37	16623258	chrX.102883803.119358070	loss	5	1.52
Xq24	GLUD2	6.93E-06	-2.65	2753995	chrX.119455338.122209332	loss	5	1.51
Xq25	XPNPEP2	6.87E-08	-3.81	7869983	chrX.128566292.129127918	loss	5	1.51

A region showing frequent gain was detected at 1q32.1 and a single gene (PCTK3) showed increased expression in 7/14 tumors. This gene mapped to region defined by the larger region of CN gain that was detected in a fewer number of tumors and not the frequently amplified region defined by the occurrence in 18/24 tumors defined by the copy number analysis. Interestingly, MUC1 also showed a 5-fold increase in expression and mapped to a region on chromosome 1q22 (1: 145583178-160740537) in 16/24 tumors in the copy number analysis and 6/14 tumors in the integrated analysis.

Frequently deleted regions defined by copy number analysis included 8p where 3 large contiguous regions were identified. With the incorporation of expression data, the regions are further refined, for example loss at 8p23.3 (8:21953232-27595453) was detected in 5-14/24 tumors used for the integration analysis. Three genes mapping to within this region included 2 unknown ESTs and the EBF2 gene which showed 4.5 fold decrease in expression levels. This region contains 873 genes. Three genes, DEFA1, DEFA3 and LOC728358, map to a refined region in 8p23.1 (8:6789275-6889920) and the ANGPT2 gene maps an adjacent region (8:6342789-6444367) and all show decreases expression in the tumor samples. The DCL1 gene showed a 6.7 fold decrease in expression and maps to region showing copy number loss in 6/14 tumors at 8p22 (8.11614644-13945615). This is the only gene showing decreased expression out of a possible 311 mapping to this region of 8p22 demonstrating how this approach can highlight potential tumorigenesis driver genes from large regions of CNA.

The novel gain identified at 5p15.33 contained a single gene where the 3' end mapped -into the highest region of overlap and the 5' end into the region showing gain in fewer samples. PLEKHG4B (pleckstrin homology domain-containing family G member 4B) is a member of the pleckstrin homology domain-containing family members which stimulate the exchange of guanyl nucleotides associated with a GTPase of the Rho family http://www.ncbi.nlm.nih.gov/gene.

### LOH and Gene Expression

The number of genes mapping to regions of LOH was accomplished by combining the data from the LOH and the raw gene expression data. Fifteen genes were found to map to regions of LOH that were detected in >3 samples. Table 3 shows the results of this analysis. Each individual expression value was confirmed using the standardized gene expression values described above. The only chromosome where deletion was accompanied by an LOH event was on the X chromosome, several genes showing down-regulation map to Xq24 and q25. The CDN1 and CDN3 genes on 16q22 showed copy neutral LOH in 2 samples and loss with LOH in another sample, however both genes demonstrated high levels of up-regulation. Examination of the individual expression level for the tumors showing the LOH confirmed that the expression levels were higher than the controls in these tumors. A similar situation was noted on 6p21.32 where the HSP1A1 gene shows copy neutral loss in 4 tumors but has increased expression levels.

**Table 3:** Integration of LOH and Gene Expression Data.

Cyto band	Copy Number Location	Gene Symbol	p-value	Fold-Change	# Samples with Copy-Neutral LOH	# Sampleswith loss and LOH	Mean of Samples with loss and LOH	State
1p13.2	chr1.111970656.112340133	C1orf183	8.2E-06	-2.22	2	1	1.53	CN-LOH
1p13.2	chr1.111970656.112340133	KCND3	1.0E-03	-3.75	2	1	1.53	CN-LOH
1p22.2	chr1.89334399.89586461	GBP4	5.1E-06	-2.13	2	1	1.57	CN-LOH
6p21.32	chr6.31698877.32259200	HSPA1A /// HSPA1B	1.4E-03	4.12	4			CN-LOH
12q24.12	chr12.109960751.111501415	ALDH2	3.8E-05	-11.38	4			CN-LOH
16q12.1	chr16.45091910.46733079	PHKB	5.0E-04	-2.06	7			CN-LOH
16q22.1	chr16.67206157.67268948	CDH3	5.0E-04	6.72	2	1	1.44	CN-LOH
16q22.1	chr16.67268948.67273787	CDH3	5.0E-04	6.72	2	1	1.49	CN-LOH
16q22.1	chr16.67273787.67334399	CDH3	5.0E-04	6.72	2	1	1.49	CN-LOH
16q22.1	chr16.67273787.67334399	CDH1	4.1E-09	43.24	2	1	1.49	CN-LOH
16q22.1	chr16.67334399.67396764	CDH1	4.1E-09	43.24	2	1	1.49	CN-LOH
19q13.41	chr19.57065800.60767150	ZNF331	1.2E-04	-4.34	3			CN-LOH
19q13.41	chr19.57065800.60767150	MYADM	7.5E-07	-3.21	3			CN-LOH
22q12.1	chr22.26560066.27655923	TTC28	9.8E-08	-5.33	3			CN-LOH
22q12.1	chr22.26560066.27655923	XBP1	3.4E-04	4.50	3			CN-LOH
Xq24	chrX.118031377.119358070	LONRF3	2.4E-05	-6.96	0	3	1.48	Loss with LOH
Xq25	chrX.123198797.123430595	ODZ1	6.2E-06	-8.12	0	3	1.47	Loss with LOH
Xq25	chrX.128574610.129127918	XPNPEP2	4.6E-07	-3.33	0	3	1.48	Loss with LOH

## Discussion

### Copy Number and Gene Expression Integration

We have conducted a genome-wide survey of infiltrating ductal carcinoma using both transcript expression analyses, as well as copy number and LOH analyses and integrated the findings from these platforms. Several similar studies have recently been reported as the utility of this approach is becoming recognized. This type of analysis permits global identification of DNA copy number alterations that lead to specific mRNA transcript-associated alterations and highlights those genes that are dynamic participants in the inception and preservation of the malignant phenotype. Several of these have used platforms with less resolution than reported here [7-13]. One report that most closely approximated our methodology used the higher resolution 500K Mapping array set and Affymetrix U133A and B arrays to monitor gene expression [6]. Although our findings for CGH overlapped with the findings of Haverty and colleagues, the genes showing concurrent gene expression alteration did not always overlap. The reason for the lack of concordance can be attributed to several factors. The probe sets defining genes on the Affymetrix U133 set were reformulated in many cases for the design of U133 plus2 array, to adhere more closely to the increased annotation available in public databases such as RefSeq. The differences may also be the different analysis algorithms used by the two groups to define copy number and gene expression.

Gains on the q arm of chromosome 1 are more frequently reported, but the exact locations vary significantly between different studies. Yao et al [10] reported a 1.74 MB gain on 1q21 (1:148592826-150329171), Chin et al [11] reported two regions of gain on chromosome 1q21 (1:144220000-145870000, 153290000-154190000). Leary et al [8] reported a common CN gain at (1:149032752-149156996). Vincent-Salmon et al [9] using BAC-based array CGH reported several CN gains of 1q21 in ductal carcinomas in situ. Haverty et al [6] reported two regions of gain (1:142593000-14288000, 1:155952000-156708000). We detected a single region of copy gain on 1q21 which occupied 23 MB contiguous region from 1q21.1-q24.1 (1:143388732-166837735) which included all of the above reported regions, however the most frequently gained region was a .13 MB region (1:149604803-149739289). No genes with expression changes mapped in this smallest region of overlap, however four genes with concurrent increased expression were SV2A, LOC730631, S100A14 and MUC1. These are the only genes with increased expression from a total of 1663 genes mapping to this region. None of these genes were reported by other studies to show concurrent increased expression.

Chromosome 8q24 is another frequently amplified region in breast cancer. Naylor et al [20] using BAC-based aCGH reported that this amplicon contained at least 2 distinct regions. Haverty et al reported 11 discreet regions of copy number gain, while Chin et al [11] reported 5. We found 3 regions of copy number gain, and three genes showing concurrent up-regulation of expression; FAM83 H, RECQL4, AND KIFC2. RECQL4 has been associated with increased metastatic potential in breast tumors [21]

Haverty et al [6] reported several regions of amplification at 11q13, this was also one of the most frequently amplified regions in our data, however genes mapping to this region did not show alteration in expression using our analysis. Chromosomal gain at 17q23 has been reported by several groups [6,7,10,20] and the regions do vary somewhat between studies as do the genes showing concurrent overexpression. The current analysis did not identify any genes within the region with gene expression alterations.

Our analysis did not detect any homozygous deletions although a few infrequent ones were detected by the PennCNV analysis, the regions were very small with few SNP markers. Loss on the p arm of chromosome 8 has been frequently reported in breast cancer [22]. Decreased expression of DLC1 at 8p22 in a region with copy number loss was identified, a finding supported by Haverty and colleagues [6]. Loss of expression due to chromosomal deletion or promoter hypermethylation has been shown in breast tumors. Initial studies towards understanding the function of DLC1 were based on overexpression of the protein in different carcinoma cell lines, demonstrating inhibition of cell proliferation, migration and invasion [23]. Evidence supporting a tumor suppressive function of DLC1 was provided by Xue et al [24], who showed that knockdown of DLC1 promoted carcinogenesis of liver cells in an in vivo model. Others have shown that DLC1 loss is sufficient to promote a more migratory behavior of breast cancer cells [25]. Down regulation of ANGPT2 expression and a loss at 8p23.1 is also supported by Haverty and colleagues [6]. Over expression of this gene has been shown to degrade tumor vasculature in vivo [26] so a loss of function would be beneficial for tumor growth.

### LOH

The emergence of SNP arrays offer the ability to define simultaneously the copy number changes and LOH events occurring in a tumor, at high resolution and throughout the genome. As such, they offer a powerful and increasingly popular platform for oncogene and tumor suppressor gene discovery. Notwithstanding, there are few publications that report SNP-array based LOH. Of those available, the data have been generated using the 10K Mapping arrays [27] or have not found any consistent data [6]. Using our higher resolution platform the most frequently detected LOH event was at 16p11.2-16q12.1 two regions bordering the centromere. LOH has been reported in this region [27] using the 10K mapping platform, however the allelic ratio plot of this region and chromosome 8p11, another region showing frequent LOH were not convincing and appeared as artifacts due to a paucity of probes in the regions. Several tumors showed whole chromosome or chromosome arm loss.

The merging of the copy number data with the LOH data revealed that many of the frequently detected LOH regions were copy neutral events suggesting a duplication of the chromosome region accompanied by the loss of the corresponding homologous region, with the net result that the cell retains two copies of derived from one parental source and no copies derived from the other. Although there were regions of copy number losses that corresponded to LOH events they were not frequent occurrences. LOH with deletion occurred on 8p12 and several regions on the X chromosome. Xq25 has been reported as a region of frequent LOH [28]. The integration of LOH with transcript expression data revealed 15 genes that displayed decreased expression and mapped to regions of LOH. Two of the cadherin genes, CDH1 and CDH2 mapping to a CN-LOH region at 16p22 showed up regulation. Increased CDH3 expression has been associated with tumor aggressiveness, being a good indicator of clinical outcome. Moreover, the aberrant expression of CDH3 in breast cancer might be regulated by gene promoter hypomethylation [29]. Over expression of a gene in a CN-LOH region may indicate that the missing allele was acting in a suppressive capacity. On the other hand, CDH1 is commonly reported as downregulated in breast cancer, and thought to be methylated [30]. The reason for the discrepancy is that the probe set design for CDH1 hybridizes to multiple targets and is most likely being aberrantly called.

### Gene Expression and Pathway Analysis

Our gene expression analysis divulged a set of up and down-regulated genes that had been previously reported in breast cancer. The pathway analysis revealed a strong correlation between the gene expression data and the canonical PPARα/RXRα Activation. PPARα has not been implicated in breast cancer previously. This ligand activated transcription factor belongs to a family of nuclear receptors. PPARα and RXRα heterodimerize and subsequently bind to PPAR response elements in the promoters of the target genes inducing a wide spectrum of metabolic effects [31]. As shown in Additional file 5, Figure S1, several genes involved in the pathway show downregulation upstream of the activation of PPARα/RXR. For example, the inflammatory signal mediation shown on the left of the diagram shows the NIK is downregulated. The cJUN gene shows downregulation which inhibits the binding to NFκB and thus interferes with the binding to PPARα/RXR. The end result is downregulation of IL-6. This downregulation of genes upstream of PPARα/RXRα activation is seen for fatty acid uptake, glucose homeostatis, lipoprotein lipase metabolism, mitochondrial β-oxidation growth hormone homeostatis and vascular smooth muscle cell migration. The focus of PPARα/RXRα has been mainly its role in obesity and atherosclerosis, however recent data suggests that crosstalk between PPARα and the estrogen receptors exists through competitive binding to the estrogen response elements [31]. Several of the networks generated from our data also involved lipid biosynthesis. The Prolactin Signaling Pathway was another canonical pathway identified as being involved in our expression data. The majority of the genes were downregulated and mainly involved in the cellular proliferation arm of the Prolactin pathway.

## Conclusions

We have presented an integrated profile of primary infiltrating ductal carcinoma. The analysis of the data has shown that regions of copy number alterations often correlate with deregulation of gene expression. Our data has confirmed the copy number and gene expression data of many other studies, however when CGH and gene expression data are integrated the findings between studies are somewhat variable. Our analysis supported previous findings indicating that DLC1 at 8p22 and ANGPT2 at 8p23.1 show decreased expression and map to regions of frequent loss. We identified chromosome 5p15.33 as a novel region that was frequently amplified in the tumor tissues and suggest that the PLEKHG4B as showing increased expression. This is the first report of global LOH integration with gene expression and several genes mapping to regions of LOH were identified. The gene expression analysis highlighted genes that are downregulated in the PPARα/RXRα Activation Pathway.

## Abbreviations

CNA: copy number alteration; IDC: infiltrating (invasive) ductal carcinoma; CGH: comparative genomic hybridization; aCGH: array-based comparative genomic hybridization; SNPs: single nucleotide polymorphisms; CEPH: Centre Etude du Polmorphisme Humain; PCA: principle components analysis; ANOVA: analysis of variance; BRLMM: Bayesian Robust Linear Model with Mahalanobis distance classifier; HMM: Hidden Markov Model; HET rate: heterozygosity rate; DEG: differentially expressed genes; IPA: Ingenuity Pathways Analysis; SLR: signal log ratio.

## Competing interests

The authors declare that they have no competing interests.

## Authors' contributions

JR and JL performed all microarray experiments including CGH and gene expression. LS aided in the initial analysis of the data. LH designed the study, performed the final analyses and drafted the manuscript. All authors read and approved the final manuscript.

## Pre-publication history

The pre-publication history for this paper can be accessed here:

http://www.biomedcentral.com/1471-2407/10/460/prepub

## Supplementary Material

Additional file 1 **Table S1: Copy Number Data**. Data was generated using genomic segmentation fragments identified contained >10 SNPS in >5 samples. Most of the fragments are contiguous stretches but are defined by the number of samples. Average from segmentation defines the average of the copy numbers detected on each tumor. Click here for file

Additional file 2 **Table S2: LOH Data**. Data was generated using HMM, fragments identified contained >10 SNPs in >3 samples. Click here for file

Additional file 3 **Table S3: PennCNV Analysis**. Twenty two samples were analyzed using PennCNV algorithm. Total fluorescent intensity signals from both alleles at each SNP (log R ratio, LRR) are calculated as well as the relative ratio of the fluorescent signals between the two alleles (B allele frequency BAF) to generate copy number states. Click here for file

Additional file 4 **Table S4: Gene Expression Data**. Comparison of 16 IDC samples and 4 control breast tissues. The data was analyzed using a 2-way ANOVA and the list was generated using an Benjamini-Hochberg FDR of 0.05 and expression alterations >2-fold increases or decreases. Click here for file

Additional file 5 **Figure S1: PPARα/RXRα Activation Pathway**. Gene expression data was imported into Ingenuity Pathyway Analysis. This pathway was identified as the canonical pathway with the highest number of member from the target gene expression data. All functional aspects of the pathway have members that show down regulation in the list of target genes generated by the comparison of tumors to normal samples. Click here for file

Additional file 6 **Table S5: Copy number and Gene Expression Integration**. The full data of copy number and genes showing concurrent loss/gain is presented. The tumors are identified that show the loss or gain/amplification and the expression value for each control and tumor sample are presented. These expression values have been converted to standardized gene expression values for control and tumor samples. These were obtained by averaging expression values for each probe set across all tumors so that the average is 0. Negative numbers indicate that the gene expression value is lower than the average and positive ones indicate higher expression. Click here for file
