# Supplementary figures and images for: Integration of transcript expression, copy number and LOH analysis of infiltrating ductal carcinoma of the breast
Source: BMC Cancer. 2010 Aug 27;10:460. doi: 10.1186/1471-2407-10-460 (PMC2939551; doi:10.1186/1471-2407-10-460)

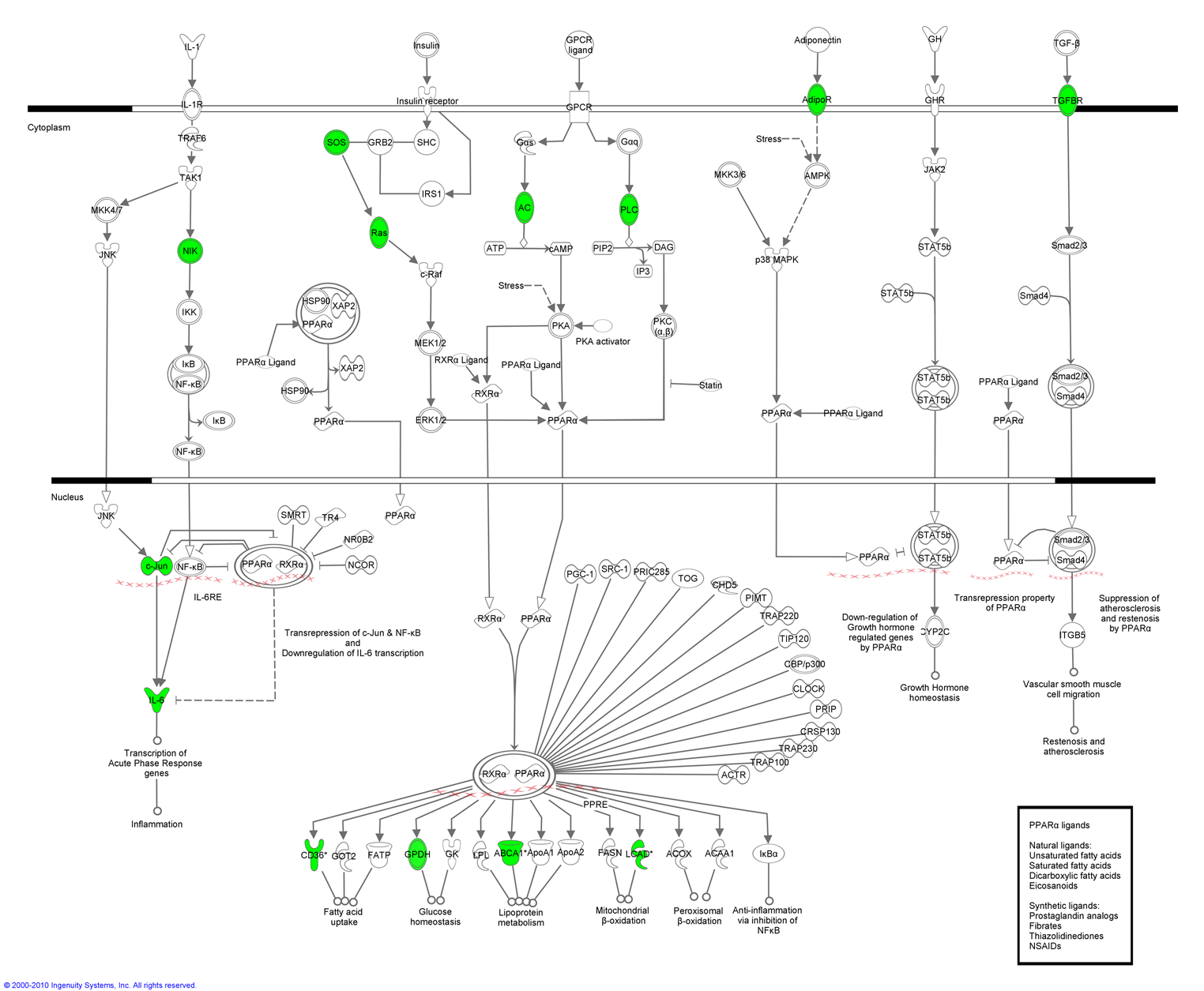

Supplement: Additional file 5 — Figure S1: PPARα/RXRα Activation Pathway. Gene expression data was imported into Ingenuity Pathyway Analysis. This pathway was identified as the canonical pathway with the highest number of member from the target gene expression data. All functional aspects of the pathway have members that show down regulation in the list of target genes generated by the comparison of tumors to normal samples. [file 1471-2407-10-460-S5.TIFF]
